# Supplementary material for: A New HIV-1 K28E32-Reverse Transcriptase Variant Associated with the Rapid Expansion of CRF07_BC among Men Who Have Sex with Men
Source: Microbiol Spectr. 2022 Oct 10;10(5):e02545-22. doi: 10.1128/spectrum.02545-22 (PMC9604004; doi:10.1128/spectrum.02545-22)
Supplement: Supplemental file 1 — Supplemental material. Download spectrum.02545-22-s0001.pdf, PDF file, 0.7 MB [file spectrum.02545-22-s0001.pdf]

1

Supplementary materials for

A new HIV-1 $K_{28}E_{32}$-RT variant associated with the rapid expansion of CRF07_BC among men who have sex with men

Jingwan Han[1,#], Yan-Heng Zhou[2,3,4,#], Yingying Ma[2], Guoxin Zhu[1], Dong Zhang[1], Bo Zhu[1], Tong Cheng[5], Lanfeng Wang[5], Jian-Hua Wang[4], Lin Li[1]*, Chiyu Zhang[2]*

Table S1. The frequencies of the wild type, $K_{28}E_{32}$ variant, and other variants among B, C and CRF01_AE subtypes.

Variants: amino acid feature at five specific sites	**Subtypes or CRFs** (n, %)					
	**A**	**B**	**C**	**D**	**CRF01_AE**	**CRF02_AG**
Wild type: E-K-EK-T	1349 (48.18%)	26633(78.35%)	7758 (71.98%)	835 (78.55%)	5457(86.07%)	1072 (63.02%)
The $K_{28}E_{32}$ variant: **K-E-VQ-S**	0	0	0	0	0	0
Intermediate (MUT-1):K-E-EK-T	11 (0.39%)	166 (0.49%)	58 (0.54%)	3 (0.28%)	82 (1.29%)	9 (0.53%)
Intermediate (MUT-2):E-K-VQ-T	0	2 (0.01%)	1 (0.01%)	0	1 (0.02%)	0
Intermediate (MUT-3):E-K-EK-S	3 (0.11%)	738 (2.17%)	88 (0.82%)	35 (3.29%)	9 (0.14%)	1 (0.06%)
Intermediate (MUT-4):K-E-VQ-T	0	0	0	0	1 (0.02%)	0
Intermediate (MUT-5):K-E-EK-S	0	0	1(0.01%)	0	1 (0.02%)	0
Intermediate (MUT-6):E-K-VQ-S	0	1 (0.003%)	0	0	1 (0.02%)	0
Others*	1437 (51.32%)	6452 (18.98%)	2872 (26.65%)	190(17.87%)	788 (12.42%)	619 (36.39%)
Total	2800	33992	10778	1063	6340	1701

* The others include any variants different from the wild type and above mentioned variants, as well as those that are unable to be translated due to the presence of degenerate bases. The subtype A contains all sub-subtypes A1, A2, A3, A4, A6, A7, and A8.

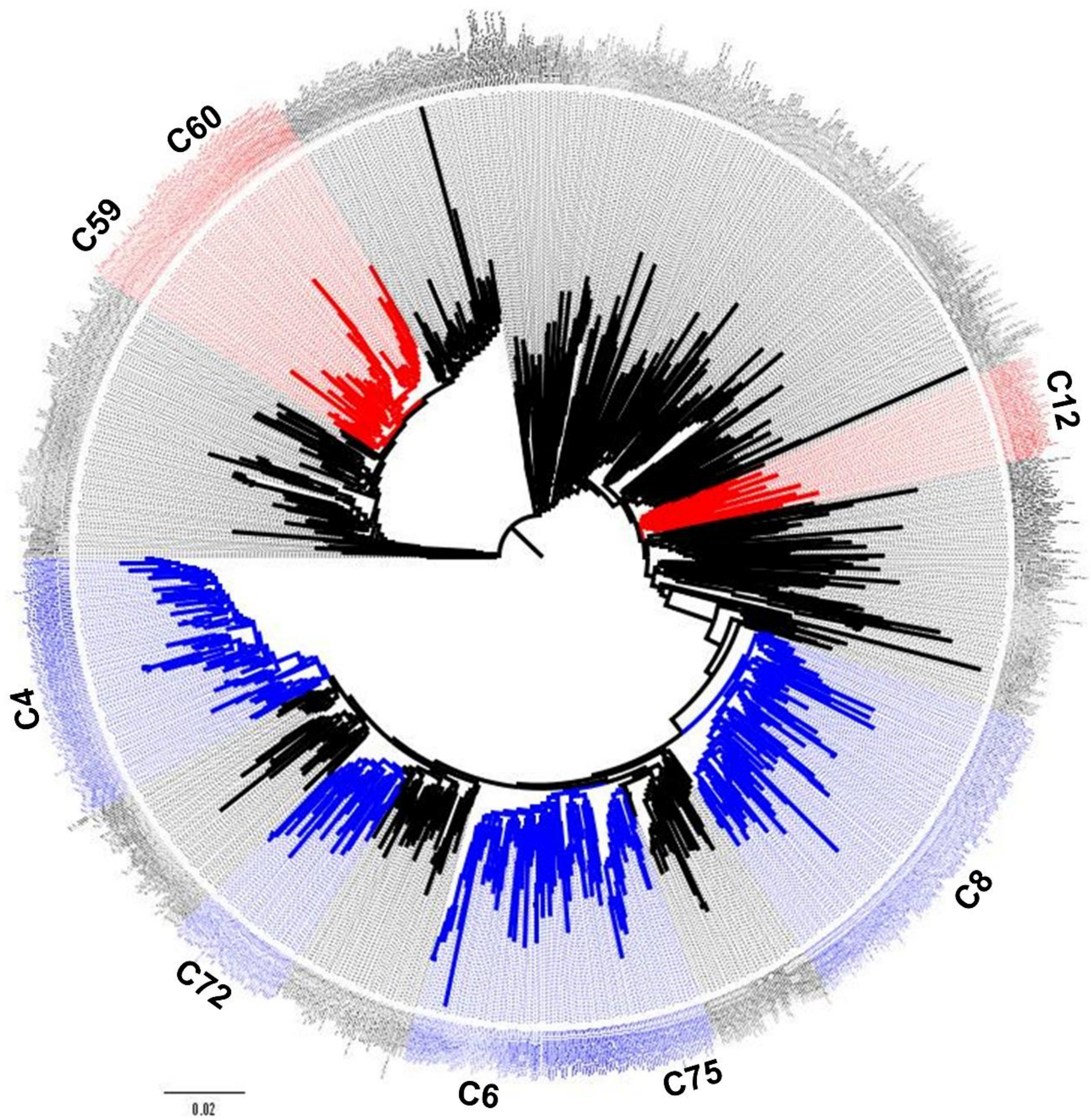

Figure S1. Maximum-likelihood (ML) phylogenetic tree of HIV-1 CRF07_BC RT coding sequences. A total of 1195 sequences from CRF07_BC in China during 1997-2013 were subjected to construct the ML tree by using FastTree program with generalized time-reversible (GTR) model. The stability of the nodes was assessed by bootstrap analysis with 1,000 replications. All the sequences were downloaded from HIV database and every sequence was from a different patient. The vast majority of the sequences were derived from plasma, few were from PBMC, and no sequences were derived from tissue. All sequences were obtained by bulk sequencing, rather than single-genome sequences. Because no outgroup references were included, the tree was rooted by the midpoint method. The red branches mark the sequences within three larger clusters (C12, C59 and C60), which mainly consist of the WT ($E_{28}K_{32}$) strains. The blue branches highlight the sequences within clusters C4, C6, C8, C72 and C75, which mainly consist of the K28E32 variant. All remaining sequences are shown in black branches, including those forming small clusters of less than 20 sequences, and those not forming clusters. C, cluster.

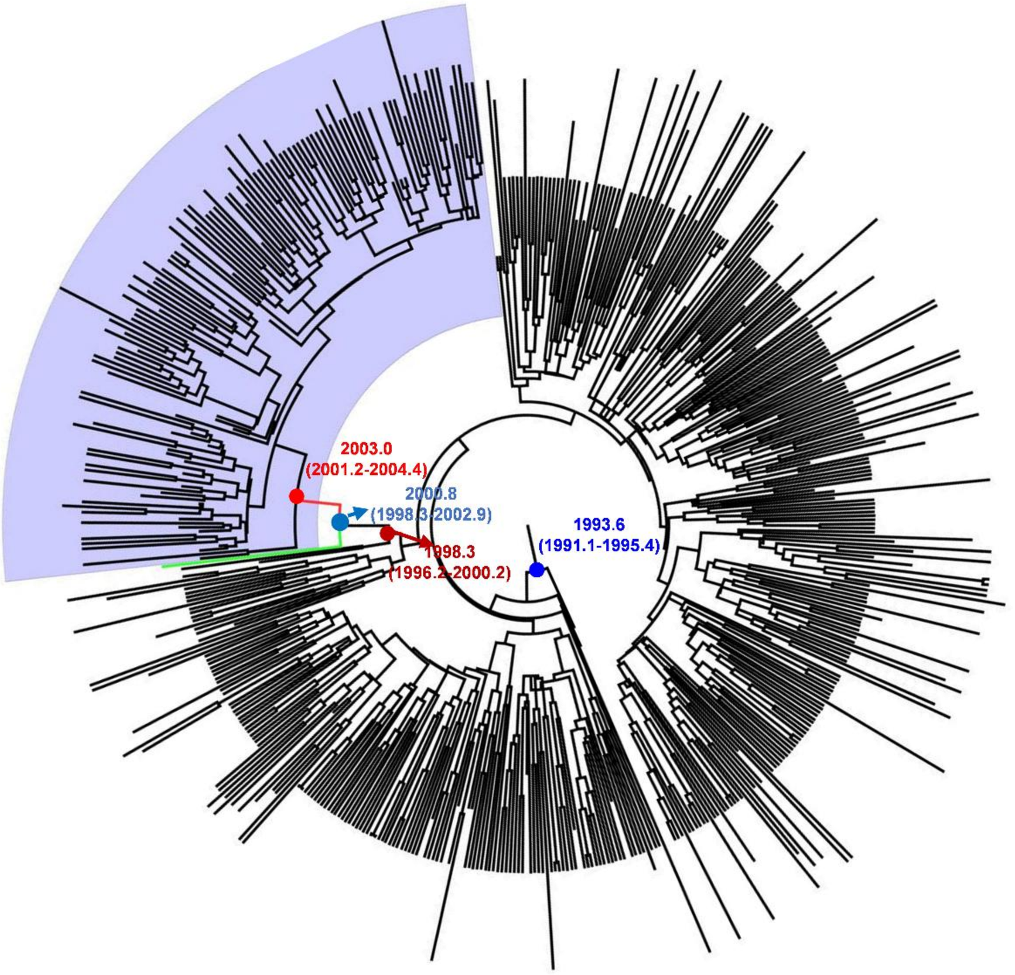

Figure S2. Maximum clade credibility (MCC) trees of the HIV-1 CRF07_BC. The $K_{28}E_{32}$ variant clade was highlighted by sky blue shadow. The evolutionary intermediate was highlighted by a green branch. The tMRCA are shown at corresponding nodes.

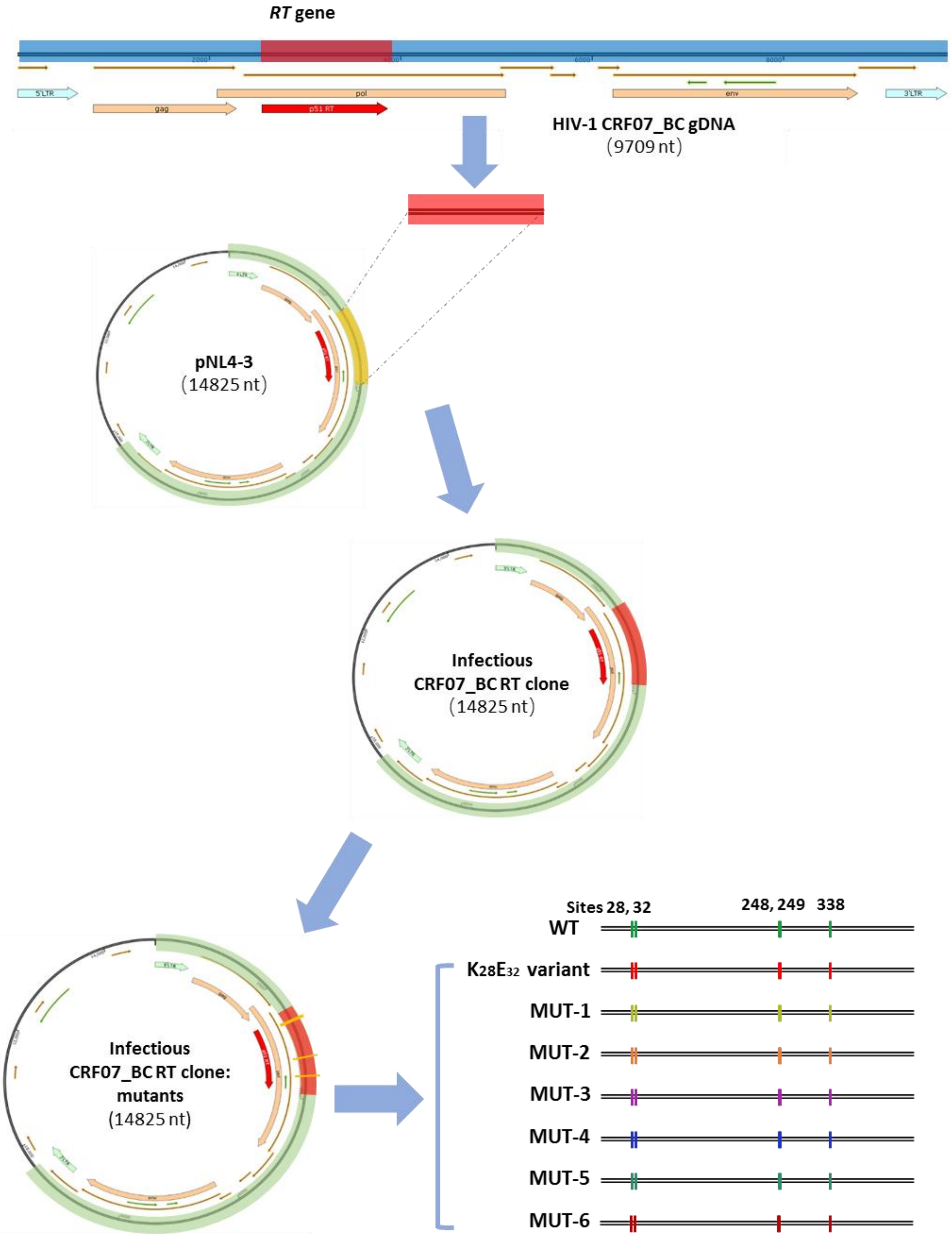

Figure S3. Construction of various CRF07_BC RT clones. The information of six mutants is provided in Tables S1 and 2.
